# Improved Lightweight Convolutional Neural Network for Finger Vein Recognition System

**DOI:** 10.3390/bioengineering10080919

**Published:** 2023-08-03

**Authors:** Chih-Hsien Hsia, Liang-Ying Ke, Sheng-Tao Chen

**Affiliations:** 1 Department of Computer Science and Information Engineering, National Ilan University, Yilan County 26047, Taiwan; r1143001@ems.niu.edu.tw; 2 Department of Business Administration, Chaoyang University of Technology, Taichung City 413310, Taiwan; 3 Department of Avionics Engineering, Republic of China Air Force Academy, Kaohsiung City 82047, Taiwan

**Keywords:** biometrics, finger vein recognition, human–machine interactions, lightweight networks

## Abstract

Computer vision (CV) technology and convolutional neural networks (CNNs) demonstrate superior feature extraction capabilities in the field of bioengineering. However, during the capturing process of finger-vein images, translation can cause a decline in the accuracy rate of the model, making it challenging to apply CNNs to real-time and highly accurate finger-vein recognition in various real-world environments. Moreover, despite CNNs’ high accuracy, CNNs require many parameters, and existing research has confirmed their lack of shift-invariant features. Based on these considerations, this study introduces an improved lightweight convolutional neural network (ILCNN) for finger vein recognition. The proposed model incorporates a diverse branch block (DBB), adaptive polyphase sampling (APS), and coordinate attention mechanism (CoAM) with the aim of improving the model’s performance in accurately identifying finger vein features. To evaluate the effectiveness of the model in finger vein recognition, we employed the finger-vein by university sains malaysia (FV-USM) and PLUSVein dorsal-palmar finger-vein (PLUSVein-FV3) public database for analysis and comparative evaluation with recent research methodologies. The experimental results indicate that the finger vein recognition model proposed in this study achieves an impressive recognition accuracy rate of 99.82% and 95.90% on the FV-USM and PLUSVein-FV3 public databases, respectively, while utilizing just 1.23 million parameters. Moreover, compared to the finger vein recognition approaches proposed in previous studies, the ILCNN introduced in this work demonstrated superior performance.

## 1. Introduction

With the advancement of computer vision (CV) technology, biometric-feature-based identification systems have emerged. Among these, the use of vein features for identity recognition has gained substantial attention and is regarded as a highly promising market trend. Moreover, as information technology continues to progress, the importance of personal data has increased, leading to the demand for more effective protection methods. Biometric recognition, which leverages the unique physiological or behavioral characteristics of individuals for identity recognition, has gained considerable attention. Hsia et al. [1] indicated four primary characteristics of biometrics: (1) Universality: biological characteristics, such as fingerprints, irises, and finger veins, are universally present in individuals. (2) Distinctiveness: each individual possesses unique biological characteristics, even twins, which make them suitable for identity recognition. (3) Permanence: the biological characteristics of individuals should remain stable over time without significant changes. (4) Collectability: the measurement of biological characteristics can be easily and conveniently performed using sensors. The inherent biological characteristics provide a reliable solution for identity recognition, causing a growing research focus in this field. Note that the security of biometric recognition methods may vary depending on the specific region from which the biological characteristics are acquired. Chen et al. [2] further classified the acquisition regions into two categories: (1) external biological characteristics, including fingerprints, irises, and faces. (2) Internal biological characteristics, such as finger and palm veins. Recently, identity recognition based on external biological characteristics has achieved significant development and found applications in mobile payments, smartphones, and access control systems. However, compared to internal biological characteristics, external features are more susceptible to environmental factors, which can degrade the model’s accuracy in biometric recognition. The following section describes the common issues encountered in the biometric recognition of external biological characteristics.

During fingerprint and palm print recognition, the presence of wounds, oil, and stains on the fingers can easily cause the capture of unstable biological characteristics by sensors, thereby affecting the model’s accuracy of fingerprint recognition [3]. Iris recognition requires expensive infrared cameras to acquire iris biological characteristics, and the brightness of lighting during this process can cause discomfort to the user’s eyes [1]. Facial recognition is highly sensitive to variations in user gender, occlusion, head pose, illumination, and facial expressions, which can decrease identity recognition [4,5,6]. Therefore, the external biological characteristics utilized in biometric recognition are unstable because they are easily affected by environmental factors. However, the veins exhibit stable internal biological characteristics. Vein features are concealed beneath the skin surface, making it challenging to steal or forge them, and veins do not undergo significant texture deterioration or change with age. Therefore, veins are better suited for identity recognition than external biometric features. However, vein-based biometric recognition has some limitations, mainly related to the approach used to acquire vein images. Owing to the limitations of low-cost near-infrared (NIR) cameras, users can be easily affected by the illumination environment when using a NIR camera to acquire finger vein images. This can result in overexposure or insufficient brightness, leading to missing vein features in the captured images. Additionally, when users perform actions such as rotating or moving their fingers during the acquisition of vein images, it often causes variations in the infrared illumination on the fingers, thereby altering the vein patterns. Consequently, when vein images are influenced by the issues, the resulting vein features can differ, making the model prone to identity recognition errors.

To address the aforementioned issues with finger vein images and simultaneously lightweight the model for practical deployment of finger vein recognition, we propose an improved lightweight convolutional neural network (ILCNN) architecture constructed by stacking diverse branch residual blocks (DBRB). The DBRB enable the model to transform into a more complex architecture during the training phase, allowing it to learn richer and more diverse feature representations. By equivalently transforming the model architecture into a simplified architecture through a function, we achieve lightweight while preserving the original model’s performance for vein recognition. However, to further enhance the model’s ability to extract subtle vein features and ensure consistency in feature extraction for translated images, this study incorporates coordinate attention mechanism (CoAM) and adaptive polyphase sampling (APS) into the DBRB residual model. This facilitates the extraction of important local features through CoAM, enabling the model to learn significant identity features among different samples. Simultaneously, APS is utilized to maintain the translational invariance of the model’s architecture after employing strided convolutional layers or max pooling, ensuring stability in vein-based identity recognition. Finally, this research introduces the elastic margin loss (EML) to minimize intra-class differences and maximize inter-class differences in the model. By normalizing weights and features in the classification layer, the proposed loss function reduces the variations among samples of the same class. Moreover, the margin obtained from a Gaussian distribution is used to amplify inter-class differences between different categories, further improving the discriminative capability of the model for vein-based identification. Overall, the main contributions of this work can be summarized as follows:

- This study proposes a lightweight model architecture, ILCNN, with only 1.23 million parameters, for finger vein recognition. The proposed architecture enables finger vein recognition in real-world scenarios with reduced parameter count, computational complexity, and inference time. Experimental results demonstrate that the lightweight model architecture outperforms other methods in terms of parameter count, computational complexity, and inference time.
- In this study, we introduce DBRB for finger vein recognition, which allows the model’s architecture to be equivalently transformed through a function, enabling the learning of richer and diverse feature maps at different scales. Experimental results indicate that the proposed DBRB effectively enhance the accuracy of finger vein recognition models in identity recognition.
- This study validates the effectiveness and generalization of the proposed lightweight model architecture by employing the finger-vein by university sains malaysia (FV-USM) and PLUSVein dorsal-palmar finger-vein (PLUSVein-FV3) databases. Furthermore, in-depth investigations are conducted to explore the impact of DBRB modules and the EML function on the proposed ILCNN model. Experimental results demonstrate that the proposed ILCNN model exhibits superior identity recognition capabilities compared to other methods across various finger vein databases.

## 2. Related Works

Previous studies have proposed methods to address the aforementioned challenges for finger vein recognition. Therefore, in the following description, we provide a brief summary of previous finger vein recognition research. Zhang et al. [7] introduced a joint Bayesian framework based on partial least squares discriminant analysis (PLS-DA) for finger vein recognition. However, their method utilized a histogram of oriented lines (HOL) as the descriptor for finger vein images, resulting in high computational complexity. This limitation makes the method susceptible to device constraints and difficult to apply in real-world environments. Zhang et al. [8] proposed a histogram of oriented physiological Gabor responses (HOPGR) as a representation of finger vein patterns. By utilizing Gabor filters, this method effectively addresses the finger tilting issue in finger vein images. However, it may degrade identity recognition when fingers are bent or subjected to longitudinal finger rotation. In [9] proposed adaptive Gabor convolutional neural networks (AGCNN) for finger vein recognition, aiming to overcome the high parameter count and computational complexity commonly associated with neural networks. However, the model still employed three linear layers as classifiers, contributing to a large number of parameters and computational burden, making it challenging to converge on finger vein databases. Zhang et al. [10] introduced a joint Bayesian model that combines finger vein region of interest (ROI) images with soft biometric features for finger vein recognition. By incorporating finger contour as auxiliary information, this method addresses issues related to finger rotation and displacement. However, finger vein image databases often exhibit numerous classes with a limited number of samples per class, potentially leading to insufficient training data for the auxiliary information input branch to effectively explore the association between primary features and auxiliary information. Liu et al. [11] proposed a novel convolutional neural networks (CNNs) architecture for finger vein recognition comprising two branches: a trunk branch and a soft mask branch. The trunk branch utilizes residual units to extract finger-vein features, whereas the soft-mask branch employs an hourglass network to extract global finger-vein features. Subsequently, the results predicted by the soft mask branch are transformed using a sigmoid function into the range [0, 1] and multiplied by the features from the trunk branch to implement an attention mechanism. However, the architecture of the model is complex, resulting in the number of model parameters exceeding five million, which may limit its deployment in real-world applications because of device constraints. Zhong et al. [12] used partial MobileNetV2 as the backbone model and performed a simple feature transformation to convert the output features from 7 × 7 × 160 to 1 × 1 × 512. The finger vein features were enhanced using automatic color enhancement (ACE) on the input images to further improve the recognition rate. However, padding the input images to squares using their own features can introduce more complex feature patterns owing to feature translation, making convergence difficult and resulting in decreased recognition rates. Chai et al. [13,14] utilized a partial MobileNetV2 architecture as a backbone model for extracting vein features. In [13], they transformed feature maps from 4 × 12 × 96 to 2 × 2 × 1024 using consecutively stacked convolutional layers and employed global average pooling (GAP) to capture spatial information while avoiding an increase in model parameters caused by fully connected layers. However, a significant increase in the channel dimensions of the model can cause a sharp increase in the model parameters, making it challenging to maintain its lightweightness. In [14], Chai et al. addressed this issue by reducing the channel dimensions during feature transformation using consecutively stacked convolutional layers in the backbone model, thereby reducing the model parameters. In addition, they applied ACE to enhance the input images and improve the generalization ability of the model. However, this model does not address the issue of vein feature translation, which potentially results in inconsistent recognition owing to variations in user operations.

## 3. Materials and Methods

**Figure 1:**
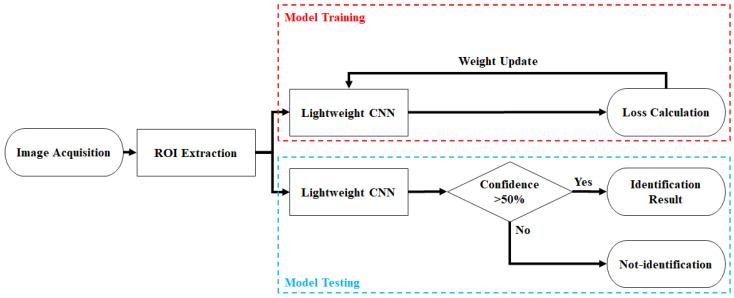
Framework of finger vein recognition system proposed in this study.

This study proposes an ILCNN model for a finger vein recognition system, which consists of two stages: training and testing, as shown in Figure 1. First, this study employs the ROI algorithm proposed in [1] to extract the ROI image from the finger vein images. Before feeding the ROI images into the model, zero padding was performed to transform the image into a square shape and satisfy the input requirements of the proposed model. In the training stage, the lightweight model architecture proposed for training is utilized, and the best-performing weight parameters are obtained by iteratively optimizing the model’s weight parameters. The results were saved as outcomes of the training phase. In the testing stage, the lightweight model optimized through training was used for finger vein recognition, identifying the individual based on the finger vein image, and computing probabilities. If the predicted probability based on the finger vein image exceeds 50%, the model recognizes it as the correct identity. Otherwise, a misclassification was considered. The following sections present a detailed introduction to the proposed architecture.

### 3.1. Model Compression

**Figure 2:**
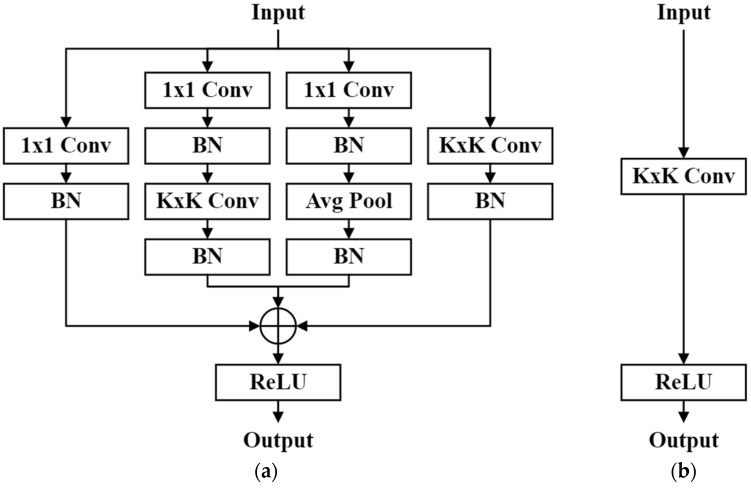
Architecture of diversity branch block. (**a**) Architecture for the training phase. (**b**) Architecture for the testing phase.

The classical CNNs architecture often achieves high accuracy in many recognition tasks, but it is accompanied by numerous parameters, making it unsuitable for real-world applications. However, with recent advancements in model compression research, various methods have been proposed to reduce the model parameters while maintaining the same accuracy, with the aim of achieving lightweight CNN models. Structural reparameterization employs a function to convert the parameters of a complex model architecture into equivalent parameters for a simplified model. This approach reduces the number of model parameters and computational complexity during the inference stage. This training strategy allows the model to benefit from rich computational resources during the training phase for optimization, and the complex model architecture enables the extraction of feature maps with different scales, further enhancing feature diversity during training. However, during the inference stage, it is necessary to deploy the model on platforms with lower computational capacity. Therefore, at this stage, the method utilizes a series of transformation functions to convert a complex model architecture into an equivalent simplified model architecture for inference. Because the model parameters before and after the transformation were equivalent, the recognition capability of the complex model architecture during the training process was equivalently transferred to the simplified model architecture. Ding et al. [15] proposed a diverse branch block (DBB) to perform structural reparameterization, as shown in Figure 2. During the training stage, a module composed of four convolutional branches replaced the original K × K convolutional layer for model training. This module extracts feature maps of different scales using branches with different complexities to enrich the feature space diversity of the model. During the inference stage, this module can be converted into a single-layer convolutional layer for model inference. Compared to using a simple K × K convolutional layer for model training, the DBB can significantly enhance a model’s feature extraction capability while maintaining the same computational complexity as a model architecture composed of K × K convolutional layers during inference. Therefore, in this study, the DBB was employed to replace all convolutional layers in the model architecture for training, while the original model architecture was utilized for inference.

### 3.2. Attention Mechanism

**Figure 3:**
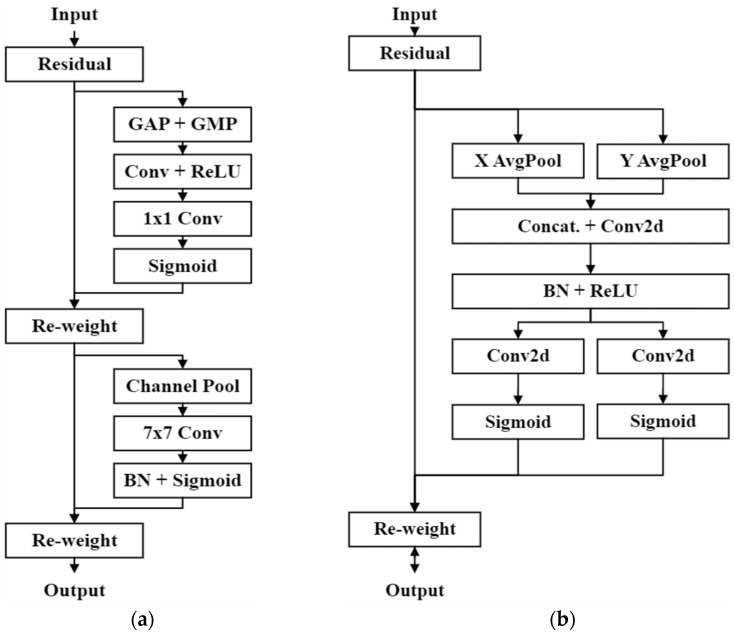
Architecture of different attention mechanisms. (**a**) CBAM architecture. (**b**) CoAM architecture.

To further enhance the capability of the model to extract effective features for finger-vein recognition, the proposed lightweight model architecture incorporates an attention mechanism. In particular, Hou et al. [16] introduced a novel attention mechanism called the CoAM. In contrast to previous methods, such as SENet [17] and CBAM [18], CoAM utilizes two average pooling layers in different directions to compress the spatial information of the feature map. The vectors from the two directions were subsequently combined by concatenation and fed into a convolutional layer to capture the channel-wise information of the feature map. During feature encoding, the shared convolutional weights ensure that information between the two direction vectors can be shared. Finally, the concatenated encoded features are divided back into two direction vectors, and each direction vector undergoes further feature transformations using independent convolutional layers. Simultaneously, the Sigmoid function is applied to transform the values of the feature vector into a range between 0 and 1 and then multiplied with the original input feature map to achieve the attention mechanism. The CoAM represents the information of the feature map through two direction vectors, where each element of the vectors indicates the presence or absence of the object of interest in the corresponding row or column. This approach allows the CoAM to perform the attention mechanism more accurately and effectively to identify the precise positions of objects. Compared with SENet and CBAM, this improved the model’s ability to recognize finger vein features. The attention mechanism architectures of the CBAM and CoAM are illustrated in Figure 3. In this study, the proposed model utilizes CoAM to facilitate the extraction of more discriminative features for finger-vein recognition, thereby improving the accuracy of identifying different identities.

### 3.3. Shift-Invariant Features

**Figure 4:**
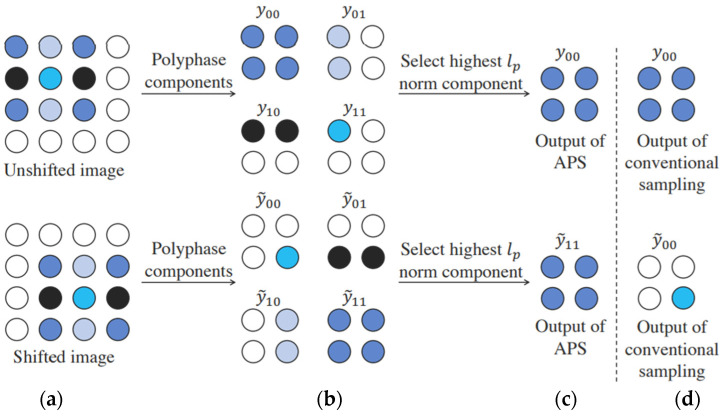
APS sampling on single-channel feature map [20]. (**a**) Feature map before and after translation. (**b**) Candidate sampling results selected by APS sampling on the feature map. (**c**) Result with the highest value after computing using $L_{p}$ normalization on the candidate samples. (**d**) Result computed using the traditional downsampling method.

CNNs are widely recognized for their ability to extract translation-invariant features. However, recent studies found that even subtle changes in input images can cause significant variations in the output results of the model [19]. In finger vein recognition tasks, when extracting the ROI from finger vein images, the captured images may undergo translation because of user operations. Therefore, image translation should be considered in designing finger vein recognition model architectures. Chaman et al. [20] introduced the adaptive polyphase sampling (APS) technique to address the problem of image translation in neural network architectures, as shown in Figure 4: APS involves down-sampling the input feature map by generating four potential candidate sampling results, known as polyphase components. Candidate sampling results were evaluated using $L_{p}$ normalization, and each candidate result was normalized using $L_{p}$ normalization. The optimal sampling result is determined by selecting the candidate result with the highest computed value. In this study, the APS sampling approach was employed to replace the original pooling layers, ensuring consistency in the extracted features and achieving stable finger vein recognition results. Moreover, $L_{p}$ normalization with a *p*-value of 2 was used to compute the optimal sampling result.

### 3.4. Margin-Based Loss Functions

In recent years, numerous studies have proposed various forms of margin-based softmax loss functions to minimize intraclass variations and maximize interclass differences to improve the accuracy of identity recognition models. Deng et al. [21] introduced an additive angular margin softmax loss (AAM-Softmax loss) method to improve the accuracy of identity recognition models. In the AAM-Softmax loss, the bias of the model’s classification layer was set to zero to simplify the representation of the softmax loss function. Using this approach, the target logits in the softmax loss function are redefined as $x_{i}W_{y_{i}}^{T}=\left\|x_{i}\right\|\left\|W_{y_{i}}\right\|\cos\left(\theta_{y_{i}}\right)$, where $\theta_{y_{i}}$ represents the angle between the weight $W_{y_{i}}$ of the model’s classification layer and the deep feature $x_{i}$. However, to address the issue of increased intra-class variation owing to inherent feature differences among training samples with the same class label, they applied $L_{2}$ normalization to both the deep features $x_{i}$ and the model’s classification layer weight $W_{y_{i}}$ to reduce the impact of class label distribution imbalance and intra-class data variation amplification. However, normalizing both deep features and weights using $L_{2}$ normalization can result in overly small outputs. Therefore, Ref. [21] multiplied the original target logit by a scaling constant s to amplify the model output. After a series of modifications, the final output of the softmax activation function depends on the cosine value of angle $\theta_{y_{i}}$, and can be expressed as $x_{i}W_{y_{i}}^{T}$ = $s\cos\left(\theta_{y_{i}}\right)$. In [21], margin penalties were added to the angle to encourage the model to maximize interclass differences and minimize intraclass variations. However, Boutros et al. [22] argued that the fixed margin penalty used in the AAM-softmax loss could hinder further convergence of the model because distinct classes have varying levels of intra- and inter-class differences. Therefore, they proposed the use of random values sampled from a Gaussian distribution as a margin penalty, offering the model increased flexibility and adaptability for learning class separability. Boutros et al. [22] proposed the EML as a modification of existing methods, particularly the $L_{EArc}$ loss function derived from the AAM-Softmax loss. The $L_{EArc}$ loss function is calculated using Equation (1).

(1) $$L_{EArc}=-\frac{1}{N}\sum_{i\in N}\log\frac{e^{s\left(\cos\left(\theta_{y_{i}}+E\left(m,\;\sigma \right)\right)\right)}}{e^{s\left(\cos\left(\theta_{y_{i}}+E\left(m,\;\sigma \right)\right)\right)}+\sum_{j=1,\;j\neq y_{i}}^{n}e^{s\cos\left(\theta_{y_{j}}\right)\;}}$$

### 3.5. Improved Lightweight Convolutional Neural Network

**Figure 5:**
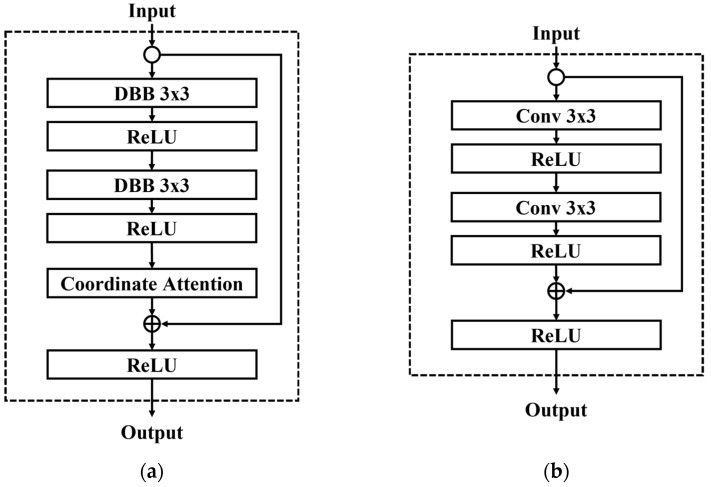
Diverse branch residual block. (**a**) DBRB proposed in this work. (**b**) Basic residual block proposed by ResNet [23].

To strike a balance between reducing the number of model parameters and maintaining high accuracy in identity recognition, this study proposes an ILCNN architecture for finger vein recognition. The ILCNN incorporates the DBRB, which is derived from the ResNet [23] residual module with specific modifications. The residual module, with its shortcut connections, effectively mitigates the issue of gradient shattering caused by the increasing depth of the model and diminished gradient correlation between neurons [24]. Consequently, models with shortcut connections often exhibit improved accuracy. In the DBRB, the DBB module replaces all convolutional layers in the original residual module and, concurrently, utilizes the CoAM and APS sampling to extract more discriminative and stable feature maps, as depicted in Figure 5a for DBRB. Ultimately, the diverse feature maps learned through the DBRB are fused with the input feature maps to enhance the model’s ability to represent vein images’ features. Therefore, this study adopts the DBRB to construct a lightweight model architecture, where the model’s complexity is enhanced during the training phase through the functions in the DBB module, enabling the model to learn effective feature representations from feature maps at different scales. During the inference phase, the model parameters can be equivalently transformed using functions, allowing lightweighting while retaining the optimized parameters. To capture more discriminative and stable features from vein images using the DBRB, this study incorporates the CoAM and APS sampling into the DBRB. By employing two different directional vectors in the CoAM, local features within the feature maps are captured, and the weighted features extracted by DBRB are used to select important vein features. Moreover, since finger vein images can undergo translation due to user behavior, the DBRB utilizes APS sampling to restore the translational invariance of the model’s architecture, even when employing operations such as strided convolutional layers and max pooling. This ensures enhanced consistency of vein features and improves the model’s stability in finger vein recognition. However, to verify the effectiveness of the proposed DBRB, we will replace it with the basic residual module proposed in the ResNet model and explore the differences between the modules. The basic residual module is shown in Figure 5b in the paper.

**Figure 6:**
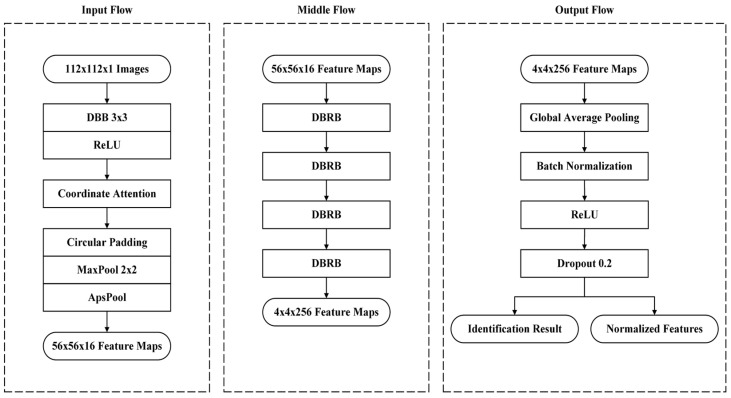
ILCNN architecture proposed in this work.

This study employed the DBRB to construct the ILCNN architecture, as illustrated in Figure 6. The model architecture was divided into three primary components: input, intermediate, and output. Each component is described in detail below.

1. Input flow
  The input flow consists of a 3 × 3 DBB, ReLU activation function, CoAM, and APS sampling. First, to address the computational constraints of the ILCNN model architecture and mitigate distortions resulting from variations in the image scale, zero padding was applied to expand the finger vein images to a square shape. Additionally, the image size is scaled to 112 before being fed into the model for feature extraction. After feature extraction using the DBB, the ReLU activation function was applied to filter the features and provide the model with the ability to capture nonlinear characteristics. Finally, this study applies consecutive CoAM and APS sampling to focus on important regions within the feature maps while reducing their size. Note that the feature maps obtained from the input pipeline were transformed from 112 × 112 × 3 to 56 × 56 × 16, with the width and height of the feature maps being half those of the input images, thereby reducing the computational burden in the subsequent stages of the model.
2. Middle flow
  After feature extraction and sampling of the input flow, four consecutive DBRBs were utilized to extract features. To mitigate the impact on the model parameters, this study avoided a significant increase in the channel dimensions of the feature maps. This is because the channel dimensions have a significant influence on the increase in the parameters. After feature extraction, the sizes of the extracted feature maps were transformed from 56 × 56 × 16 to 4 × 4 × 256. Simultaneously, the model gradually transformed the spatial information of the features into channel dimensions.
3. Output flow
  Finally, in the output flow, we applied global average pooling to further compress the spatial information of the feature maps. This approach avoids the need for direct computations using fully connected layers, resulting in a reduction in computational load. Simultaneously, we obtained a feature vector of length 256 and fed it into a fully connected layer to obtain two different types of features: normalized features using feature normalization techniques and identification results without normalization. Normalized features were employed in the computation of the loss value within the EML loss function, enabling optimization of the model weights. However, the identification results directly indicate the predicted identity class and can be utilized for subsequent evaluation metric calculations. Note that this study incorporated a dropout layer as the final layer in the output process. According to [25], placing a dropout layer before batch normalization can affect the effectiveness of batch normalization. Therefore, following the solution proposed by Li et al. [25], this study placed the dropout layer before the output layer to address this issue. This retains the advantage of dropout in enhancing the generalization capabilities, allowing the model to further improve the recognition accuracy.

## 4. Results

### 4.1. Public Database

#### 4.1.1. PLUSVein-FV3

In the PLUSVein-FV3 [26], they collected finger vein images from 60 subjects using NIR LEDs and laser sensors. Each sensor captured subsets of both the palmar (front) and dorsal (back) sides of the subjects’ fingers, resulting in a total of 7200 images, with each subset containing 1800 images. Specifically, six fingers were captured for each subject: (1) left index finger, (2) left middle finger, (3) left ring finger, (4) right index finger, (5) right middle finger, and (6) right ring finger. Each finger was considered as a separate class, resulting in a total of 360 classes in the database. The original resolution of each finger vein image was 1280 × 1024, and the authors extracted ROI, resulting in a resolution of 192 × 736 for each ROI image. To ensure a fair comparison with ILCNN, we selected only the front-side LED and laser finger vein images for model evaluation, as FV-USM database contains only front-side finger vein images. Prior to model training, we divided the ROI image datasets of the front-side LED and laser finger vein images into separate training, validation, and testing sets with a ratio of 3:1:1 for model evaluation.

#### 4.1.2. FV-USM

Asaari et al. [27] collected NIR finger vein images from four different fingers of 123 subjects in the FV-USM public database, specifically: (1) left index finger, (2) left middle finger, (3) right index finger, and (4) right middle finger. Each finger was considered as a separate class, resulting in a total of 492 classes in the database. For each finger, six NIR finger vein images were collected during separate sessions, and each person participated in a session after a two-week interval. Consequently, the database contains a total of 5904 NIR finger vein images with a resolution of 640 × 480. The dataset includes 83 males and 40 females, with ages ranging from 20 to 52 years. Before training, this study followed the preprocessing procedure described by Hsia et al. [1] to obtain an interested region of resolution 300 × 100 from the original images. The database was then divided into training, validation, and testing sets with a ratio of 4:1:1 for model evaluation.

### 4.2. Experimental Settings and Results

This study implemented finger vein recognition using an ILCNN model architecture. Note that before feeding the ROI of the finger vein images into the model, they were zero-padded to the square images and resized to match the model’s input size, ensuring that the image input met the model’s requirements. For model training, this study utilized EML as the loss function and the AdamW optimizer [28]. Regarding the hyperparameter settings, this study set the image size to 112, batch size to 32, epochs to 500, and the initial learning rate to 0.0002. In this study, all training processes are implemented using the PyTorch deep learning (DL) framework on hardware consisting of an Intel^®^ (2200 Mission College Blvd. Santa Clara, CA 95054-1549, USA). Core™ i7-12700K CPU and Nvidia RTX 3090 graphics processing unit (GPU).

In this study, an ILCNN architecture consisting of a DBB, APS sampling, and CoAM was proposed. The overall performance of the proposed model architecture was evaluated using the FV-USM and PLUSVein-FV3 public database. The correct identification rate (*CIR*) was used as the evaluation metric, which measures the security of the model. A higher *CIR* value indicates a higher level of security and a better overall system recognition performance. Therefore, the *CIR* is suitable for one-to-many finger vein recognition tasks. The calculation of the *CIR* is described in Equation (2).

(2) $$CIR=\frac{Correction\;case}{Number\;of\;total\;case}$$

To provide a comprehensive evaluation of this research, we conducted training on representative model architectures such as VGG [29], DenseNet [30], and EfficientNet [31], using the experimental environment and parameter settings as mentioned above. According to the experimental results, the proposed ILCNN model achieved *CIR* of 95.90% and 93.52% on images captured by the LED and Laser sensors, respectively, in the PLUSVein-FV3 dataset. Compared to other methods proposed in previous studies, the ILCNN model demonstrated superior *CIR* performance, as shown in Table 1. While DenseNet and EfficientNet are capable of enhancing model recognition performance by efficiently extracting image features on the ImageNet dataset and achieving impressive accuracy, they encounter difficulties in training when applied to finger vein recognition. Due to the limited number of samples in the finger vein dataset, these model architectures struggle to effectively train their parameters, resulting in diminished capabilities for identity recognition. Furthermore, to further validate the feasibility of the ILCNN model in finger vein recognition, this study conducted an evaluation using the FV-USM dataset. The ILCNN model achieved a high *CIR* of 99.82% with a low model parameters of 1.23 million on the FV-USM dataset. When compared to previously proposed finger vein models, the ILCNN model presented in this research demonstrated superior *CIR* performance with significantly fewer model parameters, as shown in Table 2. LFVRN_CE+ACE [12] attempts to pad finger vein images with square shapes using the vein image's own information, enabling identity recognition through texture features. However, due to the complexity of these texture features, the model faces challenges in accurately recognizing intricate patterns while maintaining lightweight model. Notably, the VGG-16 model with model parameters of 136.28 million achieved only 86.16% *CIR* on the FV-USM dataset. The high model parameters of this model makes it difficult to train effectively with the limited samples in the finger vein dataset, leading to issues with generalization. These results further validate that on a limited finger vein dataset, model parameters could lead to a decrease in finger vein recognition performance.

To investigate the impact of different methods in the proposed ILCNN model on finger vein recognition, we conducted an ablation study to assess their effectiveness. The incorporation of the DDB and the residual structure in the DBRB allows the model to preserve the original features while extracting features from different scales, thereby enriching the diversity of learned features. The experimental results demonstrate that incorporating the DBRB into the finger vein recognition model effectively captures the texture features in finger vein images, resulting in a 2.02% increase in *CIR* for finger vein recognition, as shown in Table 3. Furthermore, in the field of face recognition, many researchers believe that the euclidean margin-based softmax loss is not suitable for learning facial features used for identity recognition. As a result, numerous studies have started exploring the angular margin-based softmax loss, leading to significant advancements in the field of face recognition. However, whether the euclidean margin-based softmax loss is applicable to finger vein recognition remains unknown. Therefore, in this study, we conducted an ablation study using the EML loss function to explore its effectiveness. As shown in the experimental results in Table 3, when applying the EML loss function to the model based on the DBRB, the model achieved further convergence and a simultaneous 0.63% improvement in *CIR* for finger vein recognition. This indicates that the angular margin-based softmax loss yields a higher *CIR* in finger vein recognition.

In this study, we propose an ILCNN lightweight model architecture for practical deployment in real-world scenarios for finger vein recognition. However, for the model to be applicable in real-world settings, it is essential to demonstrate good performance in terms of model parameters, floating-point operations per second (FLOPs), and inference time. Among these factors, the model parameters are influenced by variations in the convolutional kernel size and channel dimensions within the model architecture. A larger number of model parameters indicates a greater memory requirement for the model. However, the model parameters alone cannot assess the execution speed of the model. Therefore, in this study, we further evaluate the model using FLOPs and inference time to gain a more comprehensive understanding of its performance. Generally, FLOPs, which are used to assess the computational complexity of a model, can be indicative of the model's execution speed. However, due to the existence of various optimized convolutional operations nowadays, the computational cost of multiplication and addition operations in the model may vary, ultimately leading to an inability to determine execution time solely based on FLOPs. Therefore, in this study, it is necessary to incorporate inference time into the model evaluation for a more accurate assessment. According to the experimental results, it can be observed that the ILCNN model architecture outperforms representative model architectures from the past in terms of model parameters, FLOPs, and inference time, as shown in Table 4. This further demonstrates that the proposed ILCNN model has certain advantages compared to other methods, not only in terms of memory requirements but also in execution speed. This indicates that our approach is less susceptible to hardware limitations and can easily be applied to handheld platforms or embedded systems, aligning with the requirements of real-world environments.

## 5. Conclusions

Following years of continuous development, finger vein recognition technology has proven to be an effective and reliable biometric recognition method. However, for applications requiring one-to-many identification with strict security requirements, several challenges must be addressed. This study proposes an ILCNN model for finger vein recognition that enhances the ability to learn diverse features within a model architecture during the training phase. It can effectively capture essential global features and possesses shift-invariant capabilities, thereby addressing the instability issues commonly encountered in feature extraction. The experimental results indicate that the proposed model achieves an impressive *CIR* of 99.82% and 95.90% on the FV-USM and PLUSVein-FV3 public databases, respectively, while effectively reducing the number of model parameters, FLOPs, and execution speed. Furthermore, the performance of the proposed model surpasses that of recent research methods.

## Figures and Tables

**Table 1:** Comparison of *CIR* with other methods on PLUSVein-FV3.

Methods	*CIR* (%)	
	LED	Laser
DenseNet-121 [30]	93.06	93.19
EfficientNet-B0 [31]	95.82	92.22
**ILCNN**	**95.90**	**93.52**

**Table 2:** Comparison of *CIR* and model parameters with other methods on FV-USM.

Methods	*CIR* (%)	Params (M)
W. Liu et al. [11]	98.58	5.85
LFVRN_CE + ACE [12]	99.09	4.93
Semi-PFVN [13]	94.67	3.35
LightFVN + ACE [14]	96.17	2.65
VGG-16 [29]	86.16	136.28
DenseNet-121 [30]	93.54	7.46
EfficientNet-B0 [31]	99.70	4.64
**ILCNN**	**99.82**	**1.23**

**Table 3:** Ablation study on FV-USM dataset.

**Methods**	**DBRB**	**EML**	**CIR (%)**
**ILCNN**	w/o	w/o	97.17
	w	w/o	99.19
	w	w	**99.82**

**Table 4:** Comparison of efficiency with other methods.

Methods	Params (M)	FLOPs (G)	Inference Time (ms)	
			GPU	CPU
VGG-16 [29]	136.28	7.94	3.66	26.60
DenseNet-121 [30]	7.46	1.37	3.30	46.00
EfficientNet-B0 [31]	4.64	0.20	3.12	10.09
**ILCNN**	**1.23**	**0.19**	**2.81**	**7.38**

## Data Availability

Code is publicly available at https://github.com/liangying-Ke/Finger-vein-recognition.

## References

[1] Hsia C.-H., Liu C.-H. (2022). New hierarchical finger-vein feature extraction method for ivehicles. IEEE Sens. J..

[2] Chen Y.-Y., Hsia C.-H., Chen P.-H. (2021). Contactless multispectral palm-vein recognition with lightweight convolutional neural network. IEEE Access.

[3] Chen Y.-Y., Jhong S.-Y., Hsia C.-H., Hua K.-L. (2021). Explainable AI: A multispectral palm-vein identification system with new augmentation features. ACM Trans. Multimed. Comput. Commun. Appl..

[4] Albiero V., Krishnapriya K.S., Vangara K., Zhang K., King M.C., Bowyer K.W. Analysis of gender inequality in face recognition accuracy. Proceedings of the IEEE/CVF Winter Conference on Applications of Computer Vision Workshops.

[5] Bisogni C., Nappi M., Pero C., Ricciardi S. (2022). PIFS scheme for head pose estimation aimed at faster face recognition. IEEE Trans. Biom. Behav. Identity Sci..

[6] Peña A., Morales A., Serna I., Fierrez J., Lapedriza A. Facial expressions as a vulnerability in face recognition. Proceedings of the IEEE International Conference on Image Processing.

[7] Zhang L., Sun L., Li W., Zhang J., Cai W., Cheng C., Ning X. (2022). A joint bayesian framework based on partial least squares discriminant analysis for finger vein recognition. IEEE Sens. J..

[8] Zhang L., Li W., Ning X., Sun L., Dong X. A local descriptor with physiological characteristic for finger vein recognition. Proceedings of the International Conference on Pattern Recognition.

[9] Zhang Y., Li W., Zhang L., Ning X., Sun L., Lu Y. (2020). AGCNN: Adaptive gabor convolutional neural networks with receptive fields for vein biometric recognition. Concurr. Comput. Pract. Exp..

[10] Zhang L., Sun L., Dong X., Yu L., Li W., Ning X. An efficient joint bayesian model with soft biometric traits for finger vein recognition. Proceedings of the Chinese Conference on Biometric Recognition.

[11] Liu W., Lu H., Li Y., Wang Y., Dang Y. An improved finger vein recognition model with a residual attention mechanism. Proceedings of the Chinese Conference on Biometric Recognition.

[12] Zhong Y., Li J., Chai T., Prasad S., Zhang Z. Different dimension issues in deep feature space for finger-vein recognition. Proceedings of the Chinese Conference on Biometric Recognition.

[13] Chai T., Li J., Prasad S., Lu Q., Zhang Z. (2022). Shape-driven lightweight cnn for finger-vein biometrics. J. Inf. Secur. Appl..

[14] Chai T., Li J., Wang Y., Sun G., Guo C., Zhang Z. (2022). Vascular enhancement analysis in lightweight deep feature space. Neural Process. Lett..

[15] Ding X., Zhang X., Han J., Ding G. Diverse branch block: Building a convolution as an inception-like unit. Proceedings of the IEEE/CVF Conference on Computer Vision and Pattern Recognition.

[16] Hou Q., Zhou D., Feng J. Coordinate attention for efficient mobile network design. Proceedings of the IEEE/CVF Conference on Computer Vision and Pattern Recognition.

[17] Hu J., Shen L., Sun G. Squeeze-and-excitation networks. Proceedings of the IEEE Conference on Computer Vision and Pattern Recognition.

[18] Woo S., Park J., Lee J.-Y., Kweon I.S. CBAM: Convolutional block attention module. Proceedings of the European Conference on Computer Vision.

[19] Zhang R. (2019). Making convolutional networks shift-invariant again. Int. Conf. Mach. Learn..

[20] Chaman A., Dokmanic I. Truly shift-invariant convolutional neural networks. Proceedings of the IEEE/CVF Conference on Computer Vision and Pattern Recognition.

[21] Deng J., Guo J., Xue N., Zafeiriou S. ArcFace: Additive angular margin loss for deep face recognition. Proceedings of the IEEE/CVF Conference on Computer Vision and Pattern Recognition.

[22] Boutros F., Damer N., Kirchbuchner F., Kuijper A. ElasticFace: Elastic margin loss for deep face recognition. Proceedings of the IEEE/CVF Conference on Computer Vision and Pattern Recognition Workshops.

[23] He K., Zhang X., Ren S., Sun J. Deep residual learning for image recognition. Proceedings of the IEEE Conference on Computer Vision and Pattern Recognition.

[24] Balduzzi D., Frean M., Leary L., Lewis J.P., Ma K.W.-D., McWilliams B. (2017). The shattered gradients problem: If resnets are the answer, then what is the question?. Int. Conf. Mach. Learn..

[25] Li X., Chen S., Hu X., Yang J. Understanding the disharmony between dropout and batch normalization by variance shift. Proceedings of the IEEE/CVF Conference on Computer Vision and Pattern Recognition.

[26] Asaari M.S.M., Suandi S.A., Rosdi B.A. (2014). Fusion of band limited phase only correlation and width centroid contour distance for finger based biometrics. Expert Syst. Appl..

[27] Kauba C., Prommegger B., Uhl A. The two sides of the finger—An evaluation on the recognition performance of dorsal vs. palmar finger-veins. Proceedings of the International Conference of the Biometrics Special Interest Group.

[28] Loshchilov I., Hutter F. Decoupled weight decay regularization. Proceedings of the IEEE International Conference on Learning Representations.

[29] Simonyan K., Zisserman A. Very deep convolutional networks for large-scale image recognition. Proceedings of the International Conference on Learning Representations.

[30] Huang G., Liu Z., Maaten L.V.D., Weinberger K.Q. Densely connected convolutional networks. Proceedings of the IEEE Conference on Computer Vision and Pattern Recognition.

[31] Tan M., Le Q.V. EfficientNet: Rethinking model scaling for convolutional neural networks. Proceedings of the International Conference on Machine Learning.

